# Towards continuous optimization of CRT settings: the relationship between pulmonary artery dP/dt and Left ventricular dP/dt measurements

**DOI:** 10.1007/s10840-023-01700-y

**Published:** 2023-11-22

**Authors:** Luuk H. G. A. Hopman, Sarah W. E. Baalman, Joris R. de Groot, Reinoud E. Knops, Vokko P. van Halm

**Affiliations:** 1 Department of Cardiology, Amsterdam UMC, Amsterdam, The Netherlands; 2 Amsterdam Cardiovascular Sciences, Amsterdam, The Netherlands

Cardiac resynchronization therapy (CRT) aims to improve cardiac contraction synchronization in heart failure patients[1]. However, not all patients respond favorably to this therapy, and individualized optimization of the device settings, particularly atrioventricular (AV) and interventricular (VV) timing, may maximize hemodynamic benefits in CRT patients [1].

Invasive testing, specifically the measurement of left ventricular (LV) performance using dP/dt, can be used for optimizing CRT [2]. dP/dt refers to the derivative of LV pressure (*P*) with respect to time (*t*), which can be used as a measure of ventricular contractility. Yet, a single optimization procedure may not be sufficient to provide long-term optimization, as requirements may change over time. To address this limitation, continuous monitoring of ventricular performance and constant optimization of CRT settings may offer significant benefits, both in terms of adaptation to ventricular remodeling and immediate support during acute hemodynamic changes, such as those experienced during physical activity.

The CardioMEMS™ pulmonary artery sensor (Abbott Laboratories, Abbott Park, IL, USA) is an endovascular hemodynamic monitor that can be used by healthcare professionals for early congestion detection [3]. This device offers continuous pulmonary artery pressure (PAP) data, which may also serve as a surrogate for left ventricular (LV) dP/dt measurements, enhancing the potential for ongoing CRT optimization [4]. However, it is not known how continuously assessed PAP_dP/dt_ measurements obtained with the CardioMEMS™ sensor compare to the LV_dP/dt_ reference standard. This proof-of-concept study aimed to evaluate the relationship between continuously obtained PAP_dP/dt_ measurements and invasively obtained LV_dP/dt_ measurements using a pressure wire in the context of optimizing CRT. If successful, the development of an algorithm utilizing PAP_dP/dt_ measurements could revolutionize pacing optimization. This approach, which enables continual evaluation of cardiac performance, could prove invaluable not only in traditional CRT but also in emerging pacing modalities like conduction system pacing.

This study was approved by the local institutional ethics committee (Amsterdam UMC, Amsterdam, The Netherlands). Five participants with a guideline indication for biventricular pacing underwent two implant procedures: first, a CardioMEMS™ sensor was inserted into the patients’ pulmonary artery to continuously monitor their PAP; and second, a CRT device was implanted, complete with a quadripolar LV lead, as depicted in Fig. 1A. Patients underwent invasive individual CRT optimization within 1 month after CRT implantation. While maintaining a constant heart rate by atrial pacing, invasive measurement of LV performance was carried out using a pressure catheter as part of the AV and VV optimization process. Meanwhile, the CardioMEMS™ sensor was used to record PAP waveforms continuously during all settings, enabling the automatic calculation of average PAP_dP/dt_ at 30 s intervals. Between each setting change, a waiting period was implemented to ensure a consistently stable state. Pearson correlation test was used to assess the relationship between PAP_dP/dt_ and LV_dP/dt_, and Bland–Altman analysis was employed to evaluate the agreement.

**Fig. 1:**
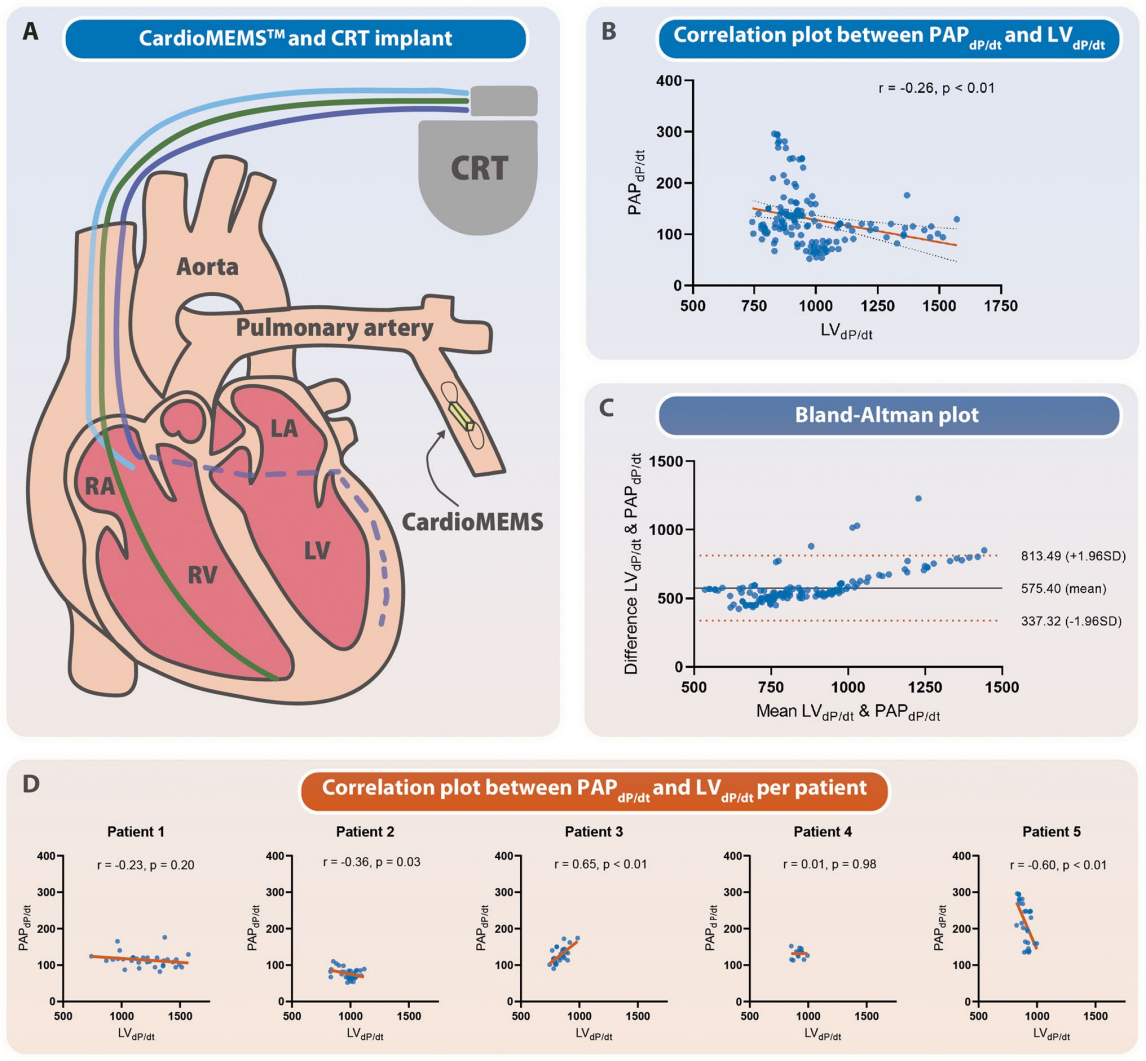
LV_dP/dt_ and PAP_dP/dt_ assessed using CardioMEMS™ during CRT optimization in CRT patients. **A** A schematic representation of the CRT and CardioMEMS™ implantation. **B** A correlation plot demonstrating the relation between LV_dP/dt_ and PAP_dP/dt_. **C** A Bland–Altman plot demonstrating the agreement between LV_dP/dt_ and PAP_dP/dt_. **D** Correlation plots demonstrating the relation between LV_dP/dt_ and PAP_dP/dt_ per patient

Five patients (100% male, mean age 75 years, ischemic etiology 60%) were included in the study). None of the patients had pulmonary hypertension and one patient had atrial fibrillation. During optimization, a total of 146 settings were tested with a mean of 29 ± 10 settings per patient. Overall, LV_dP/dt_ had a weak inverse correlation with PAP_dP/dt_ (Pearson *r* =  − 0.26, *p* < 0.01) (Fig. 1B). The Bland–Altman analysis showed a systematic bias and revealed that the variability of the differences was not constant across the range of measurements (Fig. 1C). Moreover, the direction of correlation between LV_dP/dt_ and PAP_dP/dt_ varied on a per-patient basis (Fig. 1D). In one patient, a positive relationship emerged between PAP_dP/dt_ and LV_dP/dt_. This patient, interestingly, showcased the most pronounced impairment in LV function and the least responsive LV_dP/dt_. Consequently, this observation raises the possibility of an inadequate response in PAP_dP/dt_, rendering the measurements more susceptible to changes in filling conditions.

This study aimed to evaluate the relationship between LV_dP/dt_ measurements obtained using a pressure wire and PAP_dP/dt_ measurements obtained using the CardioMEMS™ sensor in CRT patients. The findings showed a weak inverse correlation between PAP_dP/dt_ and LV_dP/dt_. The inverse relationship may likely be attributed to two main factors: reduced LV end-diastolic pressures and potential decreases in RV contractility. Our results may suggest that PAP measurements using the CardioMEMS™ sensor can provide continuous data on LV performance, for example even during exercise. However, the correlation between LV_dP/dt_ and PAP_dP/dt_ varied on a per-patient basis, indicating the complexity of this relationship may be influenced by factors such as arterial compliance, wave reflections, and response time discrepancies. Additionally, optimizing LV contractility by selecting the optimal LV_dP/dt_ not necessarily improves RV performance and therefore may not reflect a more beneficial PAP_dP/dt_ [5]. Instead, considering mean PAP values after a waiting period may hypothetically provide a better parameter for evaluating CRT response, as it allows for the harmonization of pressures and fluid shifts within the different cardiac compartments. Moreover, this methodology aligns with the design philosophy of CardioMEMS™, which is tailored for continuous, long-term patient management rather than the assessment of transient fluctuations in filling pressures.

It is important to acknowledge the study's limitations, including a small sample size, encompassing both ischemic and non-ischemic cardiomyopathy patients, which may impact the generalizability of the results. Additionally, the chosen instrument for optimization, dP/dt, does not account for changes in volumes such as cardiac preload or variations in blood pressure resulting from peripheral or pulmonic vasoconstriction. This limitation renders dP/dt optimization less robust, less reliable, and susceptible to inaccurate measurements [6]. Further studies with larger sample sizes and rigorous designs are needed to validate these findings.

In conclusion, while PAP measurements obtained using the CardioMEMS™ sensor can offer continuous monitoring of ventricular performance, the acute correlation with LV_dP/dt_ is weak and may be complex mandating more research to evaluate this potentially valuable concept.

## Data Availability

The data underlying this article will be shared on reasonable request to the corresponding author.
